# Traumatic Insanity in Its Medico-legal Relations

**Published:** 1879-12

**Authors:** Daniel R. Brower

**Affiliations:** Professor of Mental and Nervous Diseases, Woman’s Medical College; Attending Physician for Mental and Nervous Diseases, St. Joseph’s Hospital; and formerly Superintendent Eastern Lunatic Asylum of Virginia


ARTICLE V.

TRAUMATIC INSANITY IN ITS MEDICO-LEGAL RELATIONS. By DANIEL R. BROWER, M.D., Professor of Mental and Nervous Diseases, Woman's Medical College; Attending Physician for Mental and Nervous Diseases, St. Joseph's Hospital; and formerly Superintendent Eastern Lunatic Asylum of Virginia. Read before, and published by request of, the West Chicago Medical Society.

The importance of traumatic injuries of the head in the etiology of intra-cranial diseases has long been recognized. Hippocrates wrote: "Nullam capitis vulnus contemnendum," and this sentiment has been translated by Liston: "No injury of the head is too slight to be despised or too grave to be despaired of."

This axiom teaches that not only may as serious a condition as insanity, but death itself, result from an apparently very trivial scalp wound.

We had under observation during the late rebellion a case of gunshot wound of the head that in twenty days resulted in death, and *post mortem* examination showed no injury to the cranial bones, but an abscess in the brain and associated meningitis and encephalitis. This case was one doubtless of contusion of the brain, with solution of continuity of its constituent elements and without injury to the bone — the skull undergoing change of form suddenly, and the cerebral substance offering less resistance than its bony covering, extravasation and laceration of brain tissue ensued, and then the secondary condition above detailed.

The same kind of an injury may result in insanity, and the difference consists probably less in the nature of the injury and its associated phenomena, than in the inherent peculiarities of the individual.

If it should occur in one who possesses the insane temperament, instead of the rapidly progressing pathological process of acute meningitis and encephalitis, we may rather have the slow proceedings, chronic processes, low grades of inflammatory changes, or simple disturbance in dynamical relations of brain elements that result in insanity.

This suggests the important medico-legal fact that insanity is a chronic disease ; that the alteration in brain structure which precedes it requires months, years, or it may be generations for its complete development. The grandparent, for example, may have been addicted to chronic inebriety ; the parent may have, as a consequent inheritance, neuralgia, or some other of the neuroses, and the son, insanity. The disturbance in brain nutrition, beginning with the grandparent, not only undergoing transmutation in transmission, but becoming more profound, and only, in the third generation, becoming insanity.

It also follows that pathological conditions, so slow in their advancement, must have a corresponding amount of *time* as an essential factor in their recedence, and therefore no man possessing a healthy brain organization can be rendered insane by any emotion, no matter how violent it may be, and then have restored to him healthy brain action on the subsidence of the emotional disturbance. The plea of impulsive insanity, as from time to time presented in our courts (witness the Sickles, Cole and McFarland cases), has no foundation in scientific observation. It is simply the cunning device of astute lawyers and so-called experts, who either have badly-digested views on the subject, or else are impelled to their opinion by motives other than love of science.

The cranial injuries may be more severe than they seem to be. There may be, for example, fracture of the inner table of the skull, and not of the outer ; the blow may not have had sufficient force to fracture both, or its direction may have been disadvantageous, and then all that ordinary examination reveals will be a simple contusion of the scalp, and this with quite extensive fracture of the vitreous table ; then we have a case which, examined a few years afterward by the medical expert will exhibit only a cicatrix of the scalp, and the amount of injury done to the internal soft parts can only be determined by a careful study of the history of the case. This history, if the connection between the injury and a criminal act is complete, will show a neuropathic diathesis—will show that after the injury cephalalgia was frequent, that the sleep was insufficient in quantity, or else disturbed by dreams, that after a time, change was noticed in the emotional and then in the intellectual part of the mind; in rare cases both may have occurred simultaneously.

Three cases that serve to illustrate the medico-legal relations of this subject have come under our observation, two of them recently, and are here related.

*Case I.*—Capt. ——, æt. twenty-three, was wounded in the right parietal region in one of the early campaigns in Virginia; he was rendered insensible for a short time, but speedily recovered after being carried to the hospital. Examination showed a contused wound of the scalp, without any involvement of the bone. In a few days he returned to his command, apparently well. Prior to the beginning of the war, he was the junior partner of a prominent law firm in New York, remarkable for his steady and regular habits, his industry and mental brilliancy. Impelled by patriotism, he gave up his chances for preferment there, and entered the army as a private. The qualities, and these alone, which so distinguished him in New York, rapidly advanced him to a captaincy. Shortly after the injury, he began to have headache and to pass sleepless nights. About four years afterwards a change was manifest in his emotions, in that he became irritable, resentful, quarrelsome and dissolute. The attacks of headache became more severe, and were accompanied by an irresistible impulse to inebriety.

He had a wife and two children; prior to this condition, he always manifested for them the warmest attachment, but now, during the paroxysmal attacks, he treated them brutally, and yet, during the interval, his old love continuously showed itself.

These abnormal states became more frequent and violent, and finally his wife, not understanding their pathology, lost her patience, and became divorced, thus cutting him loose from his only balance-wheel.

He then left this country and went to France, and became an active member of the Commune. Here, as elsewhere, he was a leader; his outrages were conspicuous, and furnished abundant occupation for his irregular explosions of nerve force. At the close of the Commune, he escaped from France, and we last heard of him in the diamond fields of South Africa, as having escaped from jail, after a conviction for attempted murder and mail robbery.

*Case II.*—Jeremiah Kennedy, æt. thirty-nine, born in Ireland, grew up a quiet and orderly youth, affectionate and amiable in disposition, with strong religious sentiments. He entered the army at the beginning of the war, as a teamster, and in 1862, while attempting to stop a fight between two of his comrades, received, with a scale weight, several injuries to his head ; he fell senseless and remained unconscious for twenty-four hours.

About one year after this he spent a summer in the Andersonville prison, exposed to the rays of an almost tropical sun, and during this time was much troubled with headache and sleepless nights.

He returned home at the close of the war, with a manifest change in his mental traits ; he was now very quarrelsome, and subject to fits of violent and ungovernable fury. His family became afraid of him, and he conceived the delusion that they were attempting to poison him. About this time he had an attack of what was doubtless *grand mal* epilepsy. A little further along he became possessed of the delusion that other persons desired to poison him, and, as antidotes, carried about his person camphor and various roots, and frequently took doses of sweet oil. He also became convinced that numerous persons were conspiring against his life, and to defend himself against them, always carried a knife and a pistol. He also, about this time, became possessed of an intense aversion to his church and its priesthood, regarding the latter as the most active agents in the persecutions to which he thought he was subjected.

In the year 1870 he left his home and traveled from place to place, for the expressed purpose of getting rid of his tormentors; but found it impossible to escape their machinations.

During this time he had numerous attacks of *petit mal* epilepsy, and frequent attacks of epileptiform neuralgia. These attacks of neuralgia were very severe, were associated with marked pallor of the face, and were promptly relieved by inhalations of nitrite of amyl ; the pain always commenced in the head and ran down to the epigastrium.

He had at this time, also, attacks of transitory fury so intense that his friends then regarded him as dangerous and irresponsible. His wife had often spoken of his peculiar mental state, and several times had almost determined to have him adjudged insane. She was well convinced that at these times of excitement her own life was in jeopardy, while at other times he was kind and affectionate.

The case went on from bad to worse until finally he shot his wife, killing her instantly, and then attempted to kill himself. The night of the killing, before they retired, they were heard by people in an adjoining room kissing and cooing like young lovers. They were not intoxicated, although a small bottle of whisky, smelling strongly of camphor, was found in the room, about half empty. The man says he was aroused by what he supposed was some of his numerous persecutors attempting to break into his room—he seized his pistol and fired at a person walking rapidly across the room, but the whole affair seemed to him like a dream. When he came to his senses, and found that he had killed his wife, he immediately attempted to kill himself.

He was arrested and tried for murder. The plea of insanity was entered in defense, and Dr. H. M. Lyman and myself selected as experts. The foregoing history was thus gathered together. We found, on examination of the man, a cicatrix with a slight depression at the juncture of the right coronal with the sagital suture; and two other scars over the right temporal ridge without depression. The former he claimed was painful to the touch and was the starting point of the paroxysms of pain previosly mentioned.

We also ascertained that he had one paternal uncle, three cousins and a brother insane, and that his father was a person of very unstable mental organization.

Dr. Lyman and myself, with this history before us, came to the conclusion that he was an epileptic, that he had had frequent attacks of *petit mal*—of psychical epilepsy and of epileptiform neuralgia—and occasional attacks of *grand mal*—the explosions of nerve force taking place through the motor, the sensory and psychical areas of nerve action; and moreover that the killing was done while he was in a state of epileptic irresponsibility.

The prosecution endeavored to account for all his irregularities by attributing them to the effects of whisky, and in corroboration of this view the small bottle of whisky, half empty, played an important part. The judge instructed that if the insanity was the result of inebriety it was no defense — a remarkable instruction — and it resulted in a verdict no less surprising, to wit: "We, the jury, find the defendant guilty in the manner and form charged in the indictment, and fix his punishment at death by hanging. We, the jury, also find the defendant insane at the present time."

The judge granted the motion for a new trial, but Kennedy relieved the case of any further legal relations by committing suicide the day after. There was found among his effects a note, written the first day of the trial, which showed that his failure to sooner commit suicide was altogether due to a want of opportunity.

*Case III.*—Jacob Villinger, æt. fifty, came into the world with an insane heredity. His paternal grand-mother, two of his paternal uncles and two paternal cousins died insane. He manifested a degree of mental impairment at the age of puberty sufficient to earn for himself the sobriquet of "silly" and "crazy" among his companions; he grew up, however, to be a man of ordinary mental capacity, with industrious and frugal habits, raising a large family and accumulating considerable property, for one in his station.

At the age of about forty-two, while at work on the railroad track, he was struck by a passing locomotive; his left arm fractured, and his head injured sufficiently to produce a concussion of the brain, followed by loss of consciousness that continued for several days. After his recovery he complained of severe and frequent headache, had restless nights and gave evidence of a change in emotional condition, by irritability, fits of crying and dislike for society. One year after this injury he sustained another. A stair-case, he was assisting in erecting, fell, and in so doing struck him on the head and knocked him senseless.

After this second accident his mental perturbation was more manifest; he neglected his work, squandered his property and made himself penniless, and soon gave evidence of delusions; thought himself possessed of great wealth, boasted of being the third son of God, wandered about his neighborhood hatless, coatless and barefooted in midwinter. He was ardently devoted to his wife; he told several persons that she was too good for this world; that he was the third son of God and must send her to heaven; accordingly, in June 1878, in a public place, in the presence of several persons, without any warning or any evidence of passion or excitement, he shot her. She died immediately.

The plea in defence was insanity. Dr. H. M. Lyman and myself served as experts. With this history before us and with the evidence of disturbance in the functions of the nervous system, as shown by inequality of the pupils, well marked nystagmus, fibrillary twitchings of the muscles of the face, back, thorax and lower extremities, and the evidence of the jail attendants that he slept scarcely at all, ate but little and only such things as were brought from without, believing the jail food to be poisoned, we had no hesitation in saying that he was insane at the time of the homicide, and that he was insane at the time of the trial.

After this opinion was rendered, E. P. Weber, Esq., the prosecuting attorney, abandoned the case and the jury returned a verdict of insanity without leaving their seats.

